# Mycobiome structure does not affect field litter decomposition in *Eucalyptus* and *Acacia* plantations

**DOI:** 10.3389/fmicb.2023.1106422

**Published:** 2023-02-28

**Authors:** Caio T. C. C. Rachid, Fabiano C. Balieiro, Raquel S. Peixoto, Eduardo S. Fonseca, Hugo E. Jesus, Etelvino H. Novotny, Guilherme M. Chaer, Felipe M. Santos, James M. Tiedje, Alexandre S. Rosado

**Affiliations:** ^1^Instituto de Microbiologia Paulo de Góes, Universidade Federal do Rio de Janeiro, Rio de Janeiro, Brazil; ^2^Embrapa Solos, Rio de Janeiro, Brazil; ^3^Red Sea Research Center (RSRC), King Abdullah University of Science and Technology (KAUST), Thuwal, Saudi Arabia; ^4^Embrapa Agrobiologia, Seropédica, Brazil; ^5^Universidade Federal Rural do Rio de Janeiro, Seropédica, Brazil; ^6^Rede ILPF, Brasília, Brazil; ^7^Center for Microbial Ecology, Michigan State University, East Lansing, MI, United States; ^8^Computational Bioscience Research Center (CBRC), King Abdullah University of Science and Technology (KAUST), Thuwal, Saudi Arabia

**Keywords:** mycobiome, mixed plantation, litter decomposing fungi, litter decomposition, *Eucalyplus*, *Acacia mangium*

## Abstract

Mixed tree plantations have been studied because of their potential to improve biomass production, ecosystem diversity, and soil quality. One example is a mixture of *Eucalyptus* and *Acacia* trees, which is a promising strategy to improve microbial diversity and nutrient cycling in soil. We examined how a mixture of these species may influence the biochemical attributes and fungal community associated with leaf litter, and the effects on litter decomposition. We studied the litter from pure and mixed plantations, evaluating the effects of plant material and incubation site on the mycobiome and decomposition rate using litterbags incubated *in situ*. Our central hypothesis was litter fungal community would change according to incubation site, and it would interfere in litter decomposition rate. Both the plant material and the incubation locale significantly affected the litter decomposition. The origin of the litter was the main modulator of the mycobiome, with distinct communities from one plant species to another. The community changed with the incubation time but the incubation site did not influence the mycobiome community. Our data showed that litter and soil did not share the main elements of the community. Contrary to our hypothesis, the microbial community structure and diversity lacked any association with the decomposition rate. The differences in the decomposition pattern are explained basically as a function of the exchange of nitrogen compounds between the litter.

## Introduction

Planted forests are a low-cost and renewable source of raw material for industry, and reduce the pressure on native vegetation (Hinchee et al., 2009; de Carvalho Balieiro et al., 2020). These forests are an important component of the economy in many countries. In Brazil, more than 7 million hectares of forest are cultivated; these plantations are economically important and provide employment for millions of citizens (IBA, 2021). Trees of the genera *Eucalyptus* and *Pinus* are commonly used for silviculture in Brazil and are the most important sources of wood, cellulose, and charcoal (biofuel) for industry. Recently, *Acacia mangium* and *A. mearnsii* were introduced for the same uses, in addition to tannin extraction and rehabilitation of degraded areas (De Faria and Franco, 1997; Parrotta, 1999; IBA, 2021). Most of the tropical planted forests are monocultures. Despite the high productivity of monocultures, based partly on uniformity and stand-level management (de Gonçalves et al., 2013), certain nutrient disorders (de Gonçalves et al., 2013) and the occurrence of pathogens and pests (Strauss, 2001; Liu and Li, 2010) could decrease the profitability and stability of these forests.

An alternative management practice is to combine legume trees (which have a symbiotic association with nitrogen-fixing bacteria) with *Eucalyptus* spp., to improve the niche complementarity or reduce competition for available resources (Khanna, 1997; Binkley et al., 2003; Forrester et al., 2005a,b, 2006, 2007, 2010; Bristow et al., 2006; Xiang and Bauhus, 2007; Balieiro et al., 2008; Laclau et al., 2008; Voigtlaender et al., 2011). Marron and Epron (2019), analyzing 148 case studies, concluded that the overall effect of the mixture was significantly positive, i.e., 18% more productive than monocultures of non-N_2_-fixing trees. The difference was greater in temperate conditions (24% more productive) than in tropical conditions (12% more productive) and was attributed to nitrogen availability (generally lower in temperate climates). Under Brazilian conditions and based on data from experiments at five locations in Brazil and one in the Congo, the overall production of timber from mixed plantations was higher in poorer and sandy soils and where the climate was suitable for the legume (*Acacia mangium*; Santos et al., 2016).

Fungi and bacteria are ubiquitous organisms in soil and important components in silviculture. They are important for soil nutrient cycling, with a vital role in C, N, and P turnover, and their relationships with plants range from pathogenic to mutualist (Fierer, 2017; Frac et al., 2018). They can promote plant development, improving nutrient uptake and conferring stress resistance and protection against pathogens. Mycorrhizal associations are found in more than 80% of plants species, and most of these plants are dependent on these associations for efficient nutrient uptake (Tedersoo et al., 2020).

In previous studies (Rachid et al., 2013, 2015; Santos et al., 2018), our group investigated the changes in soil chemistry and their relationship to the soil microbial community in mixed and pure plantations of *Acacia mangium* and *Eucalyptus urograndis* in a field experiment. Using DGGE, DNA sequencing, and real-time PCR, we assessed the structure and abundance of different microbial groups and nitrogen-cycling genes. The results revealed a clear effect of the plant composition on the soil microbial community and nutrient turnover.

These studies indicated the need to better understand the effect of changes in soil microbial communities on nutrient cycling, especially in the context of litter decomposition, in view of recent findings that mixed plantations of *Eucalyptus* and *Acacia* (in equal numbers) had a more balanced supply of N and P *via* litterfall, together with an improved litter structure for microbial metabolism. These characteristics have a synergistic effect on decomposition rates and release of nitrogen (Santos et al., 2018).

In the present study, we addressed the question of how the litter composition and incubation site could influence the fungal community associated with the litter, and the impacts on the decomposition rate of the material. Our central hypothesis was litter fungal community would change according to incubation site, and it would interfere in litter decomposition rate. The hypothesis was constructed under the Home-field advantage theory, which refers to the phenomenon where a community performs better in its native environment as opposed to a foreign environment. In the context of litter decomposition, this could mean that certain fungal communities that are native to a specific ecosystem are better able to decompose the litter found in that ecosystem. This could be due to the fungi having evolved specific adaptations to efficiently break down the litter found in their native environment. Studies have shown that fungal communities associated with litter can vary depending on the location of the litter, and that these variations can have a significant impact on the rate and efficiency of litter decomposition. Therefore, a home-field advantage could be a possible explanation for the observed differences in fungal community composition and litter decomposition rate in different ecosystems (Lin et al., 2019; Fanin et al., 2021).

This is the first survey of the main groups of fungi that participate in decomposition of litter from mixed *Eucalyptus* and *Acacia* forestry species in Brazilian sandy soil. We designed an experiment using different litter compositions and incubation sites in pure and mixed plantations of *Acacia* and *Eucalyptus*, from the perspective of the home-field advantage hypothesis (Gholz et al., 2000), applying mid-infrared spectroscopy and DNA sequencing tools.

## Materials and methods

### Ethics statement

The samples were collected in a non-protected area and did not involve endangered or protected species according to Brazilian laws. The experiment was conducted in an experimental field of the research institution Embrapa Agrobiologia, in Seropédica, Rio de Janeiro State, Brazil (22° 46′S; 43° 41′W; 33 m elevation).

### Experimental design and site description

The decomposition study was conducted in litterbags incubated in field conditions. The experiment used material with three different origins: (i). monospecific stands of *Eucalyptus urograndis*, hereafter termed *Eucalyptus*;?(ii). monospecific stands of *Acacia mangium*, hereafter termed *Acacia*; and (iii). an intercropped plantation of these two species, hereafter termed Mix. The tree seedlings were planted with a spacing of 3 m x 3 m in plots measuring 18 m × 21 m (378 m^2^).

Leaves from each stand were collected using a net spread over the soil. The leaves were collected several times per week to prevent them from starting to decompose in the field. The material was dried in a desiccator for 24 h and stored until used to fill the litterbags (Santos et al., 2016).

For each litterbag (of a total of 185), 5 ± 0.1 g of litter was weighed and packed into litterbags measuring 25 × 25 cm with a 3 × 3 mm mesh. Each litterbag represented one experimental unit. The litterbags were left on top of the existing litter, in the field, and sampled at five different times (0, 15, 30, 60, and 180 days of incubation) for determination of the decomposition curve (with 4 replicates) and at three different times (0, 30, and 180 days) for lignin, carbon, nitrogen, and polyphenol levels (with 4 replicates) and for evaluation of the fungal community (with 3 replicates).

**Figure 1:**
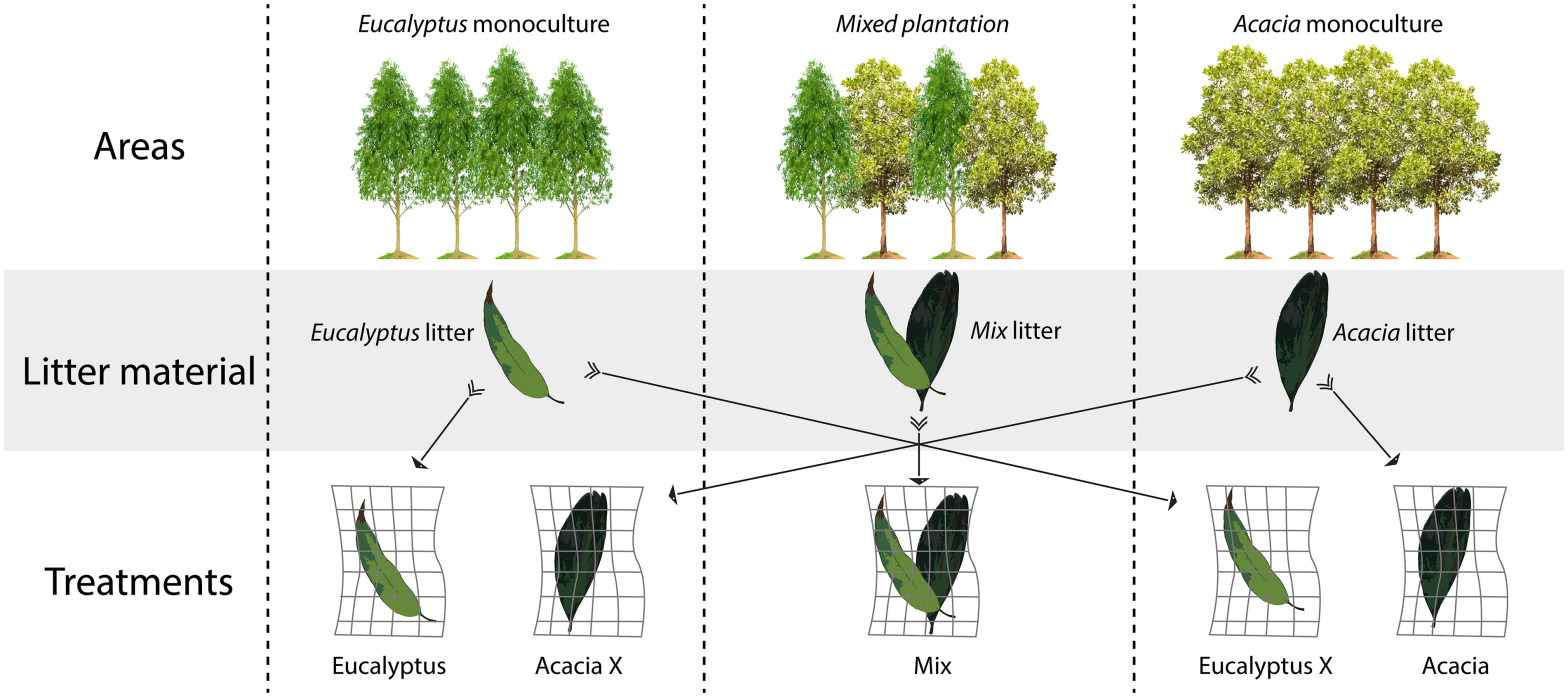
Experimental design showing the three areas, the origin of the material (litter composition), and the litterbag treatments (combination of material with incubation area).

To evaluate the effects of plant material and incubation site, we incubated each material in its own area of origin, and we also incubated the *Acacia* material in the *Eucalyptus* area and the *Eucalyptus* material in the *Acacia* area (Figure 1).

The experiment had five treatments:

- *Eucalyptus*-*Eucalyptus* litter incubated in the *Eucalyptus* monoculture area
- *Eucalyptus* X-*Eucalyptus* litter incubated in the *Acacia* monoculture area
- Mix-Mix litter incubated in the mixed plantation area (co-cultivation of *Acacia* and *Eucalyptus*).
- *Acacia*-*Acacia* litter incubated in the *Acacia* monoculture area
- *Acacia* X-*Acacia* litter incubated in the *Eucalyptus* monoculture area

A complete site description of the site was provided by Rachid et al. (2013). Briefly, the experiment was established in an area that had been left fallow for more than 15 years and was covered predominantly by *Andropogon* spp. (Poaceae), a naturally occurring grass. The soil is classified as Haplic Planosol according to the WRB/FAO system of soil classification and as Planosolo Háplico according to the Brazilian Soil Taxonomy, characterized by sandy topsoil (~90% sand), low cation-exchange capacity (CEC), and low organic-matter and nutrient contents. The regional climate is Aw (tropical with a dry winter). The mean annual precipitation is 1,250 mm, the mean daily air temperature ranges from 16°C (June and July) to 32°C (January to March), and the mean air relative humidity is 73%.

### Litter decomposition curve

To determine the decomposition pattern of the samples, litterbags were taken from the field (*n* = 4 per treatment) after 0, 15, 30, 60, and 180 days of incubation. Then, samples were oven-dried (65°C for 48 h or to constant weight) and the dry weights were determined. Because the upper layers of the forest floor would likely contaminate the litterbags with mineral mass, we determined the ash-free portion of the litter samples by heating subsamples (previously ground and homogenized) in a muffle furnace at 450°C for 5 h.

### Litter chemical characterization

The lignin concentration in litter samples was estimated using the method of acid detergent lignin (ADL; Van Soest et al., 1991). The ADL method uses 1 N sulfuric acid to solubilize sugars, starches, hemicelluloses, and some pectins, and detergents (normally Cetyl Bromide Trimethyl Ammonium, CTAB) to remove proteins. The ADL isolates mainly cellulose and lignin, with some contamination by pectin, minerals (ash), and nitrogen compounds (Van Soest et al., 1991).

The polyphenols were extracted with methanol (50%), using 1 g of litter. To prepare the standard curve, a tannic-acid solution was used. The determinations were performed in triplicate and the result expressed similarly to the method of Folin-Denis (AOAC, 1960), based on the absorbance in a spectrophotometer at 760 nm.

### Litter characterization using Fourier transform infrared spectroscopy

Ground samples were diluted in KBr (1:100) and transformed into pellets. The samples were analyzed in the mid-infrared region (4,000–400 cm^−1^) in absorbance mode, using 32 scans with a spectral resolution of 4 cm^−1^ (Song et al., 2017).

### Microbial community analysis

Litterbags were collected in the field, stored individually in plastic bags, and immediately taken to the laboratory for processing. The entire contents of each litterbag were ground in liquid nitrogen to homogenize and reduce the litter particles to powder. The resulting powder (0.2 g) was used for total DNA extraction, using a DNeasy PowerSoil DNA Isolation Kit (QIAGEN, Netherlands), according to the manufacturer’s instructions, except for the lysis step, done with FastPrep equipment (Bio 101, United States) at a frequency of 5.5 for 40 s. Each DNA extraction was done twice, and the double extraction products were combined and passed through an additional purification step to remove impurities. For this purpose, the ZR DNA Clean & Concentrator^™^ purification kit (Zymo Research Corporation, United States) was used, following the manufacturer’s instructions.

The extracted DNA was subjected to PCR amplification, targeting a fragment of the large subunit of the rRNA gene (28S rRNA), using the MID adapted primers LR0R (5′-ACCCGCTGAACTTAAGC-3′; Cubeta et al., 1991) and LR3 (5′-CCGTGTTTCAAGACGGG-3′; Vilgalys and Hester, 1990), and sequenced, as described by Rachid et al. (2015).

The raw sequences were processed with Mothur (Schloss et al., 2009), removing sequences shorter than 200 nucleotides and/or with quality lower than Q20. Then, sequences were randomly normalized to the same number (4,800 sequences per sample) and classified using the 28S database of the Ribosomal Database Project (Cole et al., 2009) to obtain the taxonomic assignment and the relative abundance of the fungal groups in each sample. The classifier was run at a 50% confidence threshold to determine the identification of the fungal community and at 0% to generate the matrix for statistical (Wang et al., 2017) analysis. All sequences that did not belong to kingdom Fungi were discarded. To increase the reliability of the results, we considered, at any taxonomic level, only the taxa represented by more than 50 sequences (summing all samples). To calculate the diversity indices, a matrix with the distribution of the genera was processed using the statistical program PAST 4.0 (Hammer et al., 2001).

In addition, we compared the fungal community of the litter with the soil fungal community described by Rachid et al. (2015), which were retrieved from the same site, in the same time frame. We compared 24 soil samples (8 from each forest stand, which were sequenced with the same primer and same method applied to the litter samples, with the 39 litter samples. The sequences are available in the NCBI Sequence Read Archive under accession number SRP033106).

### Data analysis

The statistical significance of the effects of litter origin, decomposition time, and incubation site on the C, N, polyphenol, and lignin levels, and the richness and diversity indexes were assessed using a three-way ANOVA, after testing for normality (Shapiro–Wilk W) and homoscedasticity (Levene’s test), with GraphPad Prism 7 software, excluding the Mix samples, to comprise a full factorial experiment. The spectra obtained from the litter characterization by Fourier transform infrared spectroscopy were analyzed using Principal Components Analysis (PCA), after mean center the spectra. Due to the typical light scattering of the particulate samples, it was necessary to apply an extended multiplicative scatter correction (EMSC) followed by normalizing the spectra to unitary vectors (all spectra with one standard deviation).

To explore the relationship between the fungal community of each sample and the C, N, polyphenol, and lignin levels, a matrix of fungal genera vs. samples was used as input for a NMDS with Bray–Curtis dissimilarity, with a random initial configuration, using PAST 3.2 (Hammer et al., 2001). The same procedure (without abiotic factors) was used to evaluate the relationships between the litter and soil fungal communities at the genus level.

Co-occurrence networks were constructed using the CoNet plugin version 1.1.1 (Faust and Raes, 2016) under Cytoscape version 3.8.2 (Shannon et al., 2003). Taxonomic matrixes, at genus level, were imported into the plugin, a filtering procedure was performed (matrix = count, minimum row sum: 10), and a Spearman correlation with 0.7 threshold and Fishers’ *Z* value of *p* threshold = 0.001 with Bonferroni correction was used. These edges were merged into a multi-graph with a mean score, and only edges supported by at least two methods were kept.

To evaluate the effects of the litter origin, decomposition time, and incubation site on the fungal community, we used a Three-way PERMANOVA with Bray-Curtis distance in PRIMER/PERMANOVA + software (Clarke and Gorley, 2015), excluding the Mix samples, to comprise a full factorial experiment.

To determine if the treatments had a significant effect on the specific groups of fungi, we used the blocked Indicator Species Analysis (ISA; Dufrene and Legendre, 1997) in the PC-ORD statistical package, V6.04. In ISA, we considered as significant all results with *p* < 0.05 and an indicator value (IV) higher than 70.

## Results

### Litter dynamics

**Figure 2:**
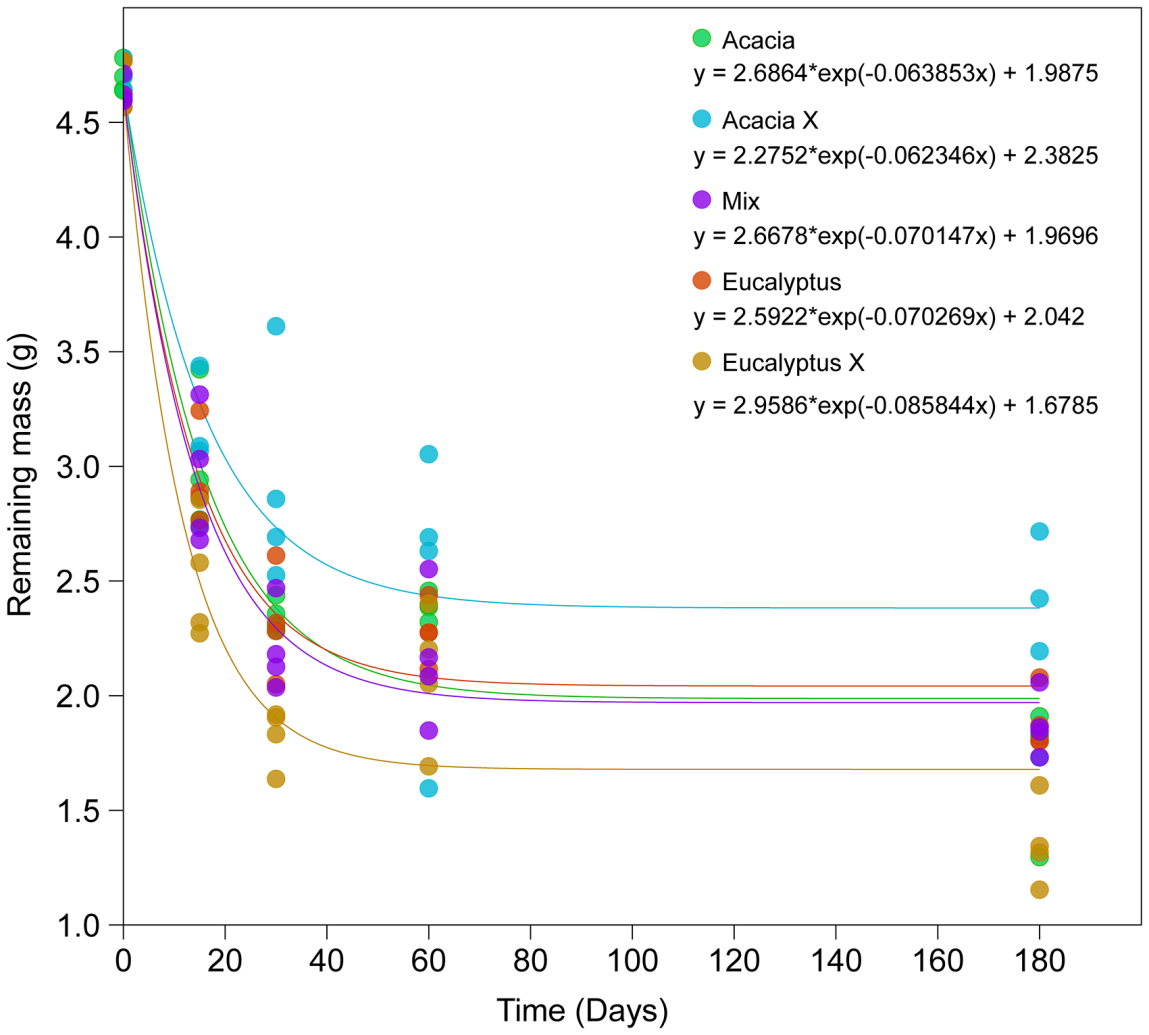
Decomposition curve of the litter in the different treatments after 0, 15, 30, 60, and 180 days. Equations are the exponential decay models for each treatment. Acacia = *Acacia* litter incubated in the *Acacia* plantation, Acacia X = *Acacia* litter incubated in the *Eucalyptus* plantation, Mix = litter from the intercropped plantation of *Eucalyptus* and *Acacia*, Eucalyptus = *Eucalyptus* litter incubated in the *Eucalyptus* plantation, and Eucalyptus X = *Eucalyptus* litter incubated in the *Acacia* plantation.

The decomposition rate was high in the first 30 days of incubation, with a mean mass loss ranging from 42% (*Acacia* X) to 64% (*Eucalyptus* X), with intermediate and very similar values among the other treatments (53–56% mass loss; Figure 2).

After 180 days, an interesting pattern was evident. The litterbags incubated at the *Acacia* site were more degraded than those incubated at the *Eucalyptus* site. However, the litterbags filled with *Eucalyptus* material were more degraded than those filled with *Acacia* material. The material in litterbags incubated at the Mix site showed intermediate levels of degradation. The factorial analysis showed influence of both plant material (two-way ANOVA, *p* = 0.02) and incubation site (two-way ANOVA, *p* < 0.01) on litter decomposition. The analysis revealed that litter (leaves) from *Eucalytpus* was more easily degraded than leaves of *Acacia*, and that litter incubated under *Acacia* trees degraded faster than under *Eucalyptus*.

**Figure 3:**
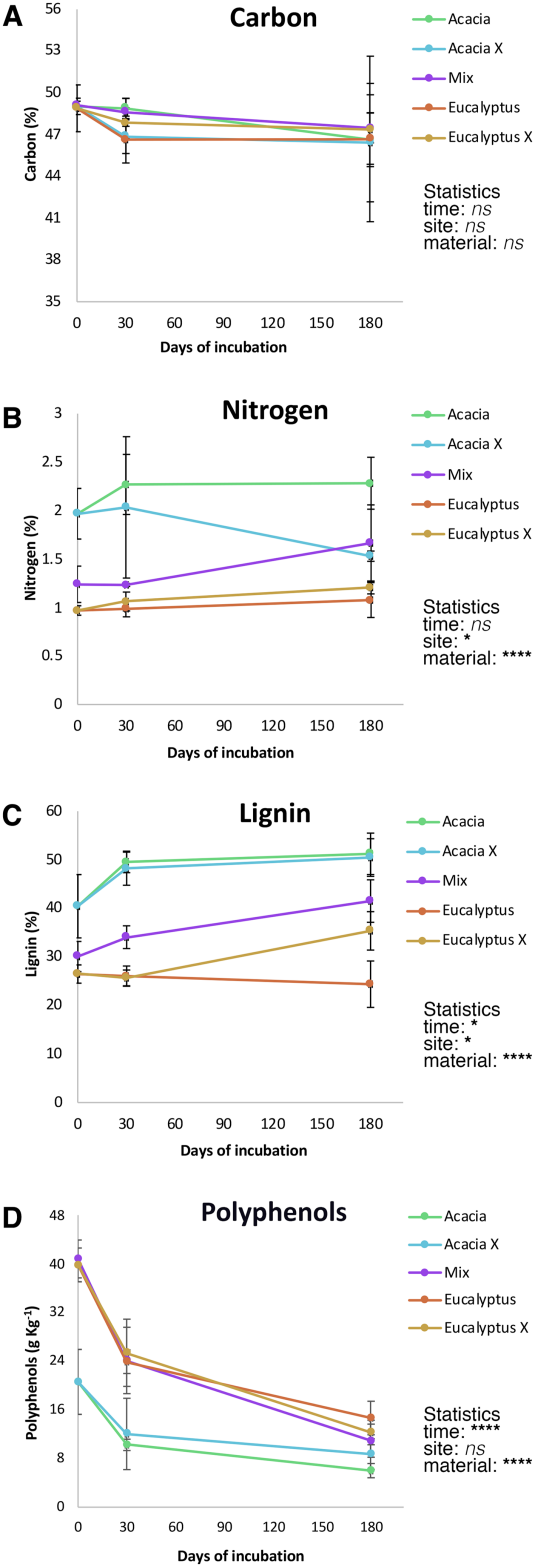
Litter biochemical dynamics during 180 days of incubation. Points represent mean values (*n* = 4); vertical bars represent one standard deviation. Statistics are based on a three-way ANOVA, *ns* = not significant, ^*^p < 0.05, and ^****^p < 0.0001. **(A)** Carbon; **(B)** Nitrogen; **(C)** Lignin; **(D)** Polyphenols. Acacia = *Acacia* litter incubated in the *Acacia* plantation, Acacia X = *Acacia* litter incubated in the *Eucalyptus* plantation, Mix = litter from the intercropped plantation of *Eucalyptus* and *Acacia*, Eucalyptus = *Eucalyptus* litter incubated in the *Eucalyptus* plantation, and Eucalyptus X = *Eucalyptus* litter incubated in the *Acacia* plantation.

Chemical characterization of the litter material (Figure 3) showed differences in nitrogen, lignin, and polyphenols (*p* < 0.0001).” Whereas the *Acacia* material showed higher nitrogen and lignin contents, the *Eucalyptus* material had higher polyphenol levels. Material from the mix treatment showed intermediate levels, as expected, for all biochemical components. Interestingly, the incubation site had a significant (*p* < 0.05) effect on the polyphenol and nitrogen contents. Litter from *Acacia* showed a marked reduction in nitrogen content when incubated at the *Eucalyptus* site, and *Eucalyptus* material showed a small increase in nitrogen content when incubated at the *Acacia* site. The polyphenol levels showed the inverse pattern.

The *Acacia*, *Eucalyptus*, and Mix material had very similar carbon contents on day 0, with a marked increase in within-treatment variability and no significant changes in any of the factors analyzed.

**Figure 4:**
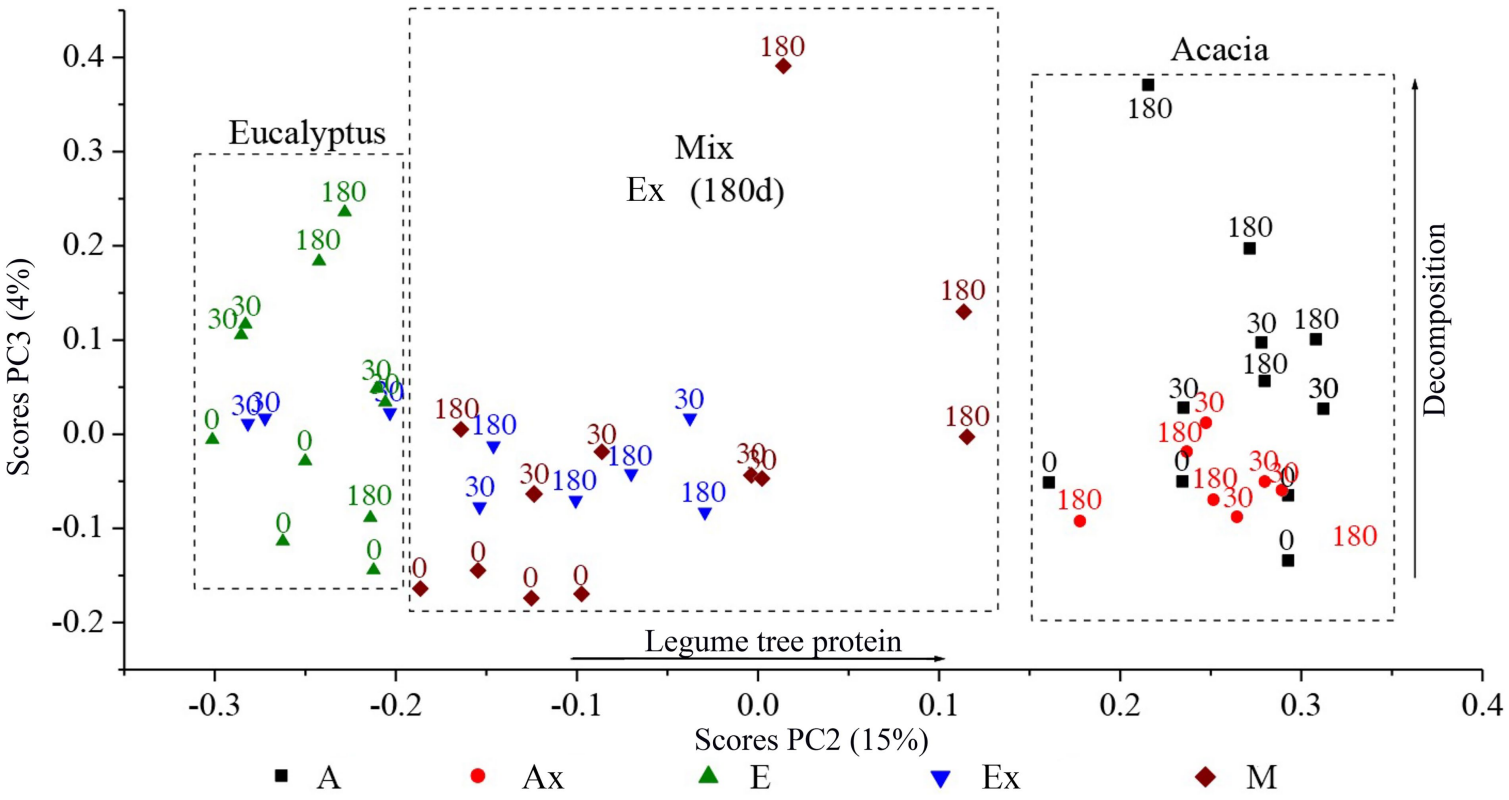
Ordination of the PC2 and PC3 of the litterbag samples in different treatments, based on the results of infrared spectroscopy. E, *Eucalyptus*; Ex, *Eucalyptus* incubated in *Acacia* stand; M, Mix; A, *Acacia*; Ax, *Acacia* incubated in *Eucalyptus* stand.

Analysis of the data from infrared spectroscopy (FTIR) by PCA showed that most of the variation in the samples (74%) was related to the inorganic compounds, with positive loadings for bands typical of Si-O (probably tectosilicate; Supplementary Figure S1), most likely related to the presence of soil particles from the area (highly sandy), and some negative loadings for bands typical of organic materials (C–H_n_). As the components from PCA are orthogonal, it is possible to isolate independent sources of variation. Thus, analysis of the other components, although representing lower total variability of the data set, allows the interpretation of the other sources of variation present in the study (PC2 and PC3; Figure 4).

As seen in Figure 4, the PC2 (15% of the total variance) captured the variability of the source material (*Eucalyptus*, *Acacia*, and mix). While PC3 (4% of the variance) captured the decomposition stage of the tested biomasses in the different stands. Both PCs presented positive loadings for proteinaceous compounds (amide bands, Supplementary Figure S1) however, the drivers were different.

The ordering of the data in the PCA, therefore, revealed that the materials were separated as a function of nitrogen content (protein), with the lowest levels found in the material from the *Eucalyptus* treatment and the highest levels in the material from the *Acacia* treatment. As expected, the Mix treatment showed intermediate N levels and trended toward *Acacia* litter after the longest incubation times, i.e., in the advanced stages of decomposition, agreeing with the N data (Figure 3B).

The exchange of materials between the incubation sites had different effects on each material. Samples incubated in the stand of origin showed a migration pattern to the upper part of the vertical axis (PC3). Samples of *Eucalyptus*, when incubated in the *Acacia* stand, trended toward the right, i.e., toward a higher N content. The *Acacia* samples incubated in the *Eucalyptus* stand remained stable in the same location relative to the vertical axis (PC3), unlike the *Acacia* samples incubated in the *Acacia* area.

### Dynamics of litter mycobiome

The fungal richness in all treatments was very similar, with around 108 different genera of fungi observed per treatment (Supplementary Figure S2A). There was no significant effect of time, site of incubation, or origin of material on the richness. However, the diversity was higher in the *Acacia* material than in the *Eucalyptus* material, regardless of the incubation site, as indicated by higher Shannon and lower dominance indexes (both *p* < 0.0001, Supplementary Figures S2B,C).

In terms of composition, the phylum Ascomycota predominated in all litterbags, comprising approximately 80% of the fungal community, regardless of the origin of the material (*Eucalyptus*, *Acacia*, or Mix), the incubation time (0, 30, or 180 days), or the incubation site (data not shown).

**Figure 5:**
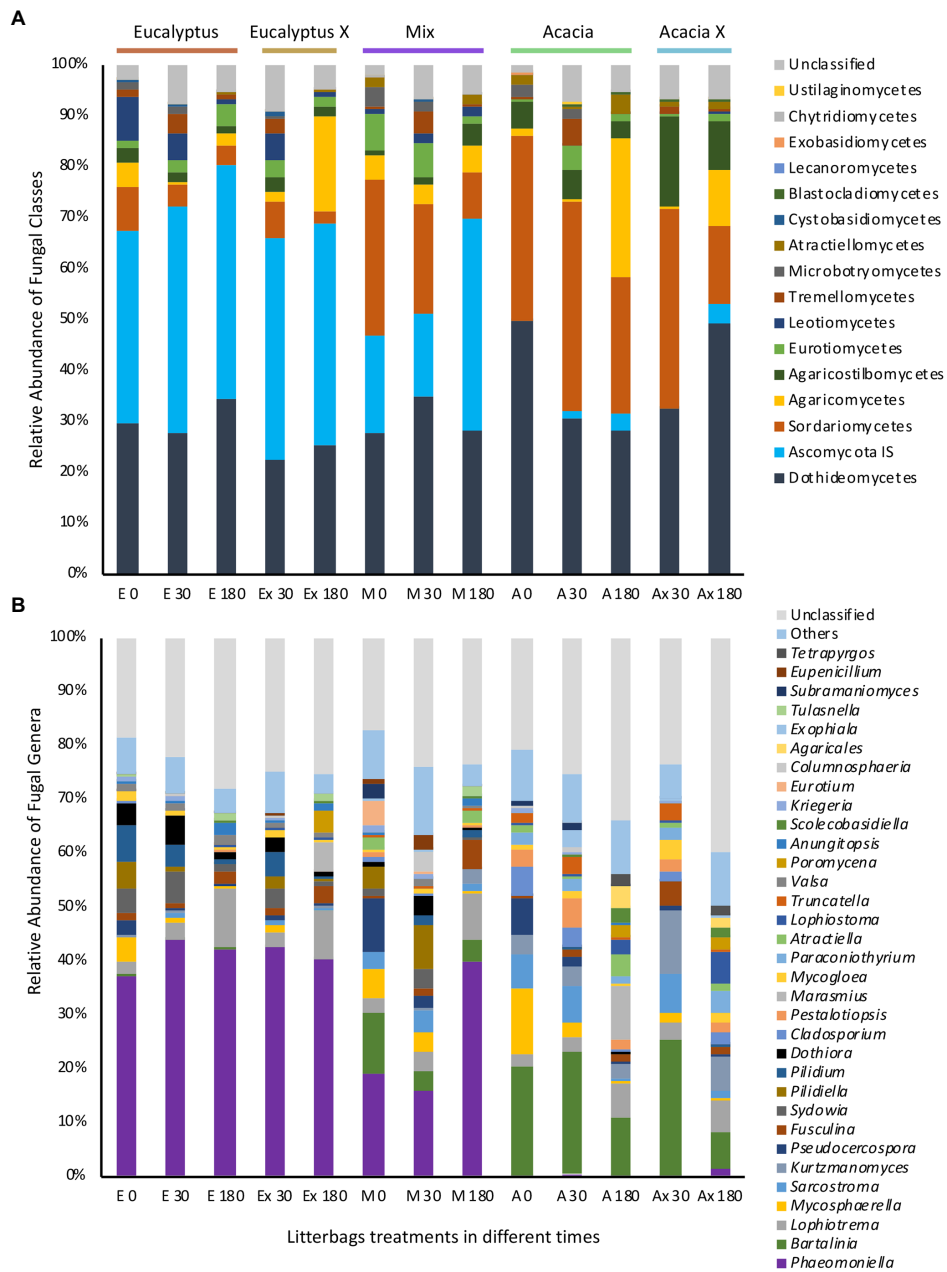
Relative mean (*n* = 3) abundance of different classes of fungi in samples of litter in different treatments. **(A)** Class level. **(B)** Genus level. On the *x* axis: initial letters represent the five treatments (E, *Eucalyptus*; Ex, *Eucalyptus* X; M, Mix; A, *Acacia*; Ax, *Acacia* X); numbers represent the incubation time in days.

The apparent uniformity of the fungal community composition of the different treatments was not apparent at the class level (Figure 5A). At this level, we detected a strong influence of the origin of the material, differentiating the treatments. Litter in all treatments had a high abundance of Dothideomycetes, which comprised about 30% of the sequences. However, the relative frequencies of two other abundant fungal taxa differed widely between the source materials (*Acacia* and *Eucalyptus*). One, Ascomycota *incertae sedis*, comprised approximately 35% of the sequences from litterbags containing *Eucalyptus* material, regardless of location and incubation time. This taxa was found in very low abundance in litterbags with *Acacia* material. The other, Sordariomycetes, was quite abundant in litterbags containing material from the *Acacia* treatment and occurred in low frequency in litterbags containing *Eucalyptus* material. The Mix treatment showed a balance of the two classes, especially at the earlier incubation times.

The most abundant genera found in Eucalyptus litter were Phaeomoniella, Lophiotrema, Pilidium, Sydowia, and Dothiora; while Bartalinia, Kurtzmanomyces, Sarcostroma, Lophiotrema, and Mycosphaerella were the most abundant in Acacia material (Figure 5B); i.e., the difference in the main genera between the two types of material was very pronounced. The blocked indicator species analysis performed to compare the composition of the fungal community based on the origin of the litter material showed that of the 60 most abundant fungal genera, 19 were significantly more abundant in Eucalyptus material and 20 were significantly more abundant in *Acacia* material (Supplementary Figure S4). Using the same approach but comparing the incubation sites, none of the genera was significantly associated with a specific location.

**Figure 6:**
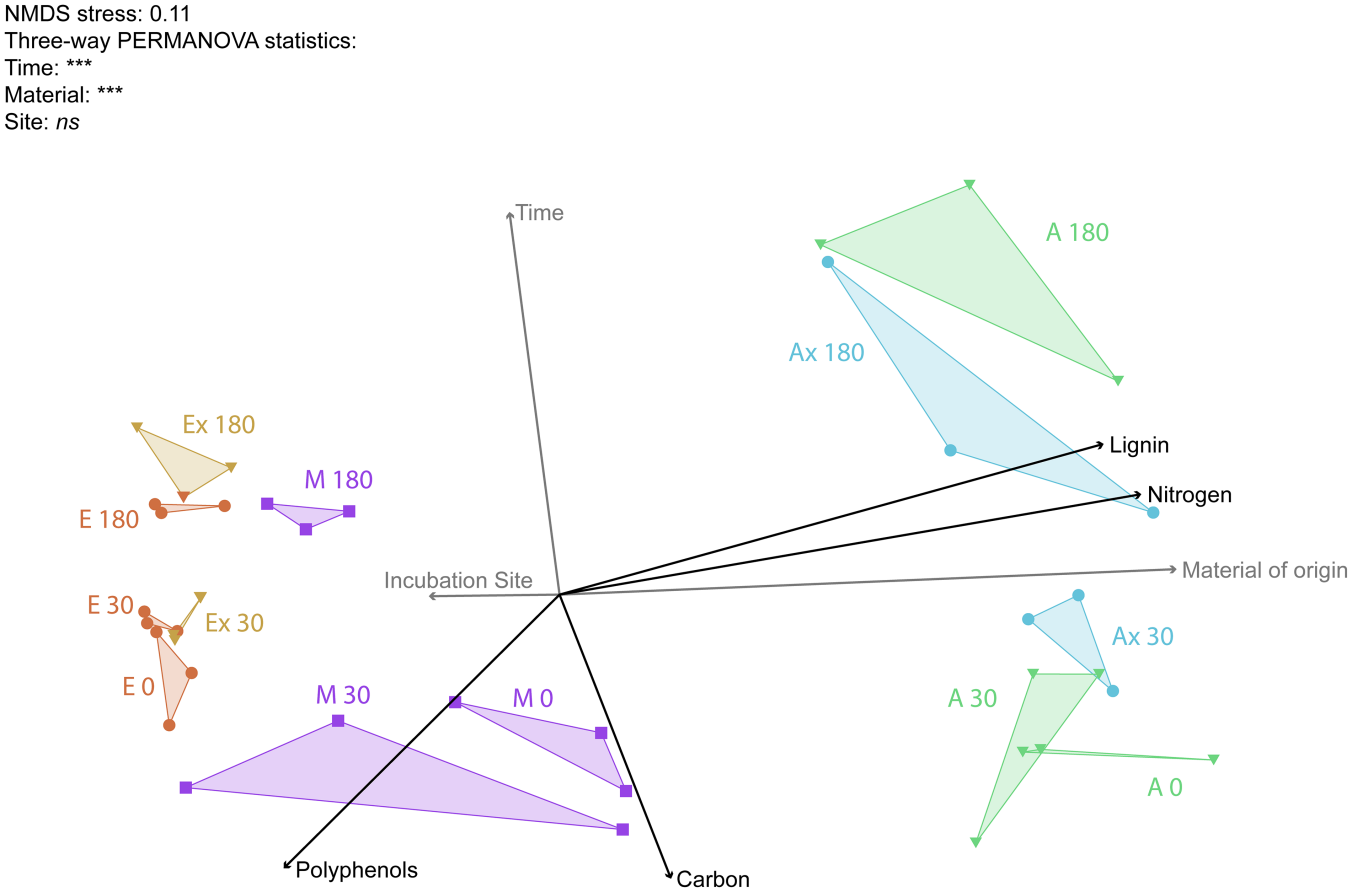
Non-metric multidimensional scaling (NMDS) ordination of fungal genera profile found in the litter samples in the different treatments. Each triangle represents a combination of the three replicates of each treatment. The initial letters represent the five treatments (E, *Eucalyptus*; Ex, *Eucalyptus* X; M, Mix; A, *Acacia*; Ax, *Acacia* X). The numbers represent the incubation time in days. The angles and the length of radiating lines indicate the direction and strength of the relationship between the chemical variables (in black) and the factors tested (gray) with the ordination scores.

Ordination of the fungal data (Figure 6) showed a clear clustering pattern based on the material of origin, with all *Eucalyptus* litterbags on the left and all *Acacia* litterbags on the right. The Mix samples showed an integration of the microbial community found for each species separately, positioned between the two other materials. Clearly, the most important driver of the fungal community was the origin of the material. The community changed gradually with time, and this difference became pronounced only in the later stages of decomposition. The incubation site did not influence the microbial community, as can be seen in the neighboring positions of the communities with the same origin but incubated at different locations. The NMDS ordination of the fungal community showed a very similar pattern to that observed in the spectroscopic data.

**Figure 7:**
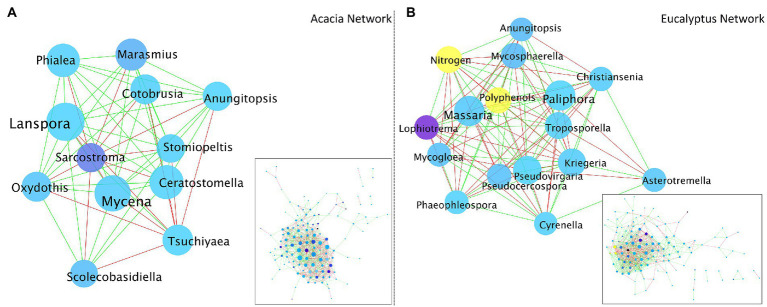
Co-occurrence network analysis based on the fungal communities of **(A)**. *Acacia* and **(B)**. *Eucalyptus* litter. Shade of node color reflects the relative abundance of a genus (darker blue indicates higher abundance). Size of node reflects the number of interactions (co-occurrence + mutual exclusion; larger node indicates more interactions). Edge colour depicts positive (green) and negative (red) correlations. Biochemical factors are colored in yellow.

We constructed a co-occurrence network analysis, one based on *Acacia* and one based on *Eucalyptus* fungal communities (Figure 7). We used this approach to explore the direct or indirect interactions between the fungal taxa coexisting in each type of litter material. The network highlighted some interesting differences between the communities, not in relation to general metrics (Supplementary Table S1), but to aspects of the main players in the network. When the two networks were filtered to display only the core nodes (those with 25 or more connections), it was apparent that the *Acacia* network was mostly supported by the relationship among a few genera, such as *Lanspora*, *Mycena*, *Ceratostomella*, and *Phialea* rather than any biochemical factor. On the other hand, for the *Eucalyptus* network, the levels of nitrogen and polyphenols were some of the most important factors contributing to the co-occurrence or mutual exclusion of the fungal community, along with the genera *Massaria*, *Paliphora*, and *Kriegeria*.

Integration of the data for the litterbag fungal community with the soil fungal community from the same site (Supplementary Figure S4) showed clearly that these two components had very distinct fungal communities. Even with the pronounced differences between the material of origin, the litterbag fungal communities resembled each other more than they resembled the fungal community in the soil where they were incubated.

## Discussion

### Decomposition of *acacia* and *eucalyptus* litter

The decomposition of litter from *Acacia mangium* and *Eucalyptus* spp. in pure and mixed plantations has been studied intensively in recent decades (Kunhamu et al., 2009; Bachega et al., 2016; Santos et al., 2018), and some results were summarized by de Carvalho Balieiro et al. (2020). According to this compilation, although litterfall deposits more N in pure and mixed plantations with N_2_-fixing trees, this does not guarantee higher litter decomposition rates in the tropics; (ii) *Acacia mangium* litter decomposes more slowly than *Eucalyptus* litter, although the leaves are high in N; (iii) the low decomposition rate of *Acacia mangium* litter has been attributed to its high lignin content and low content of non-structural carbohydrates, low molecular-weight phenols, and P, since it has a high rate of internal cycling of P; (iv) the N:P ratio tends to increase in litter in mixed plantations: *Eucalyptus* (N:*p* = 14) < legume trees (N:*p* = 25) < mixed (N:*p* = 32); litterfall contributes the most N in legume plantations, followed by mixed plantations, with the lowest N in litter in *Eucalyptus* plantations; and recycling of P tends to be lowest in mixed plantings.

The present findings concord with the literature: senescent *Eucalyptus* leaves decomposed more quickly (*k* = 0.0702 g g^−1^ d^−1^ for E and 0.0858 g g^−1^ d^−1^ for Ex) than *Acacia* leaf litter (*k* = 0.0639 g g^−1^ d^−1^ for A and 0.0624 g g^−1^ d^−1^ for Ax). When the two residues were mixed, the values of the decomposition constant were intermediate (*k* = 0.0701 g g^−1^ d^−1^ Mix), but closer to *Eucalyptus*. The more significant mass loss of the *Eucalyptus* leaf litter was due to the higher leaching of polyphenols (soluble) from these residues or to use by decomposers. Several studies are in agreement with these findings (Gholz et al., 2000; Wieder et al., 2009; Bachega et al., 2016; Santos et al., 2018).

As at the *Acacia* site the *Eucalyptus* leaf litter (Ex) decomposed faster than at its own site, we hypothesized that N (from *Acacia*) is used by fungi in the decomposition of senescent *Eucalyptus* leaves. The N from the N_2_-fixing tree partly overcame the nutrient limitation of *Eucalyptus* litter, but a labile C source was essential for decomposition. The polyphenol decay curves and the increase in N in *Eucalyptus* tissues (shown spectroscopically) attest to these findings (Figures 2, 6). The central role of nitrogen was also demonstrated in the network analysis. While nitrogen was a hub node in the *Eucalyptus* network, it was not present in the *Acacia* network, not even as a minor node. This suggests that nitrogen does not control the structure of the *Acacia* fungal community, whereas nitrogen is very important for the structure of the community associated with *Eucalyptus* litter and can control its decomposition.

At the *Eucalyptus* site the decomposition of *Acacia* material was inhibited (Figure 4), even with the abundance of dissolved C sources. Figure 3B shows a decay in the mean nitrogen content in *Acacia* X. This may indicate that the fungal community is using the available N of *Acacia* material to decompose the surrounding *Eucalyptus* litter. We also speculate that P, ordinarily low in *Acacia* leaves, was one limiting factor for decomposition (Santos et al., 2016), leading to starvation-inhibition of decomposers as proposed by Hättenschwiler et al. (2011). In other words, the *Acacia* microbial community is not able to compete with the *Eucalyptus* fungal community for P. So, for decomposition of *Acacia* litter at the *Eucalyptus* site, the nutrient limitation appears to impair the efficiency of the *Acacia* microbial community. Also, this could be an inhospitable environment for the *Acacia* fungal or bacterial decomposer community (Nuccio et al., 2013; Hoyos-Santillan et al., 2018).

### Home-field decomposition effect

The home-field advantage (HFA) theory states that plant litter decomposes faster in its own environment compared to a foreign one (40). Many authors have tested this theory, with contrasting results (Gholz et al., 2000; Ayres et al., 2009; Gießelmann et al., 2011; Bachega et al., 2016; Lin et al., 2019), in part challenging the generalization of this phenomenon to terrestrial ecosystems.

Our results are similar to the only other study with litter of these two genera (*Acacia* and *Eucalyptus*), carried out by Bachega et al. (2016). Conducted under pure and mixed plantations of *Acacia mangium* and *Eucalyptus grandis*, at a site with better soil fertility but less suitable for *Acacia*, the study refuted this theory for leaves or fine roots of the two species. For *Acacia*, the authors suggested that the time since the start of the first rotation was insufficiently long to allow the decomposers to become specialized for its litter. However, our previous results demonstrate that in the short term, soil bacterial and fungal communities differentiated and became specific for *Eucalyptus* and *Acacia* (Bini et al., 2013; Rachid et al., 2013, 2015). The microbial community of each species changes little over time, irrespective of the incubation site, which indirectly refutes the HFA. As commented by Lin et al. (2019), it “is clear that the magnitude of HFA is difficult to predict unless we discover the underlying mechanisms of the HFA effect of decomposition.”

### Mycobiome structure and diversity during decomposition

Even working with a simplified biological system (two tree species: one nitrogen-fixing tree, *Acacia*, and a non-N-fixing tree, *Eucalyptus*), Bachega et al. (2016) had difficulty in understanding the decomposition of *Acacia* and *Eucalyptus* residues within the scope of the HFA study. They recommended an approach in which the interaction between the quality of the litter and the activity of the decomposers could be explicitly analyzed; and our study attempted to go in that direction.

Curiously, in our study fungal community structure and diversity did not show any association with the decomposition rate. The number of genera, Shannon index, dominance (data not shown), and community structure were very similar in samples of the same origin and with very different decomposition rates. On the other hand, samples of different origins but with very similar decomposition rates had different microbial communities. Therefore, we also reject the HFA theory, because the structure of the fungal community in litter was not influenced by the incubation site. We also demonstrated that the dominant soil fungal community shared no members with the litter.

Our results contrast with the findings of Lin et al. (2019), who studied the HFA effects across a wide range of litter quality and forest types (broadleaf, bamboo, and conifer) using pyrosequencing. According to them, litter type was the most important variable, explaining 20.8% of the variation in the fungal community structure. Although there was no statistically significant effect of incubation site, the interaction between incubation site and litter type was the second most important driver, explaining 16.3% of the variation in the community structure. Still, our results indicate that the fungal community responsible for litter decomposition probably becomes established before the leaves fall, due to the small degree of change in the first 30 days of decomposition (period of the highest decomposition activity). The fungal community is selected by the litter source, based on specific niches, which will be driven mostly by the quality of the litter and may be colonized basically by the phyllosphere microbiome.

The apparently absence relationship between fungal community structure and litter decomposition rate can be explained by the functional redundancy of the fungal community, typical of high diverse microbial communities. In natural habitats, the high microbial richness results in different taxa presenting the same ecological niche. Thus, even when some main taxa of the community are replaced by others, the ecological role remains functional and stable (Konopka, 2009).

This study focuses on the exploration of fungal communities in relation to litter decomposition, mainly due to higher decomposition role of fungi, compared to other microorganisms (Pascoal and Cássio, 2004). However, it is important to note that the decomposition process is a complex process that is carried out by a variety of organisms, including microfauna and bacteria. Bacteria play a crucial role in the initial phase of decomposition, as they can break down labile compounds. Other microbiological attributes such as enzyme production and microorganism activity levels also play a role in the rate of litter decomposition (Pascoal and Cássio, 2004; Bani et al., 2018). While the present study found that fungal community structure did not significantly impact the rate of decomposition, the influence of other components such as bacteria and microfauna cannot be ruled out.

We conclude that both the origin of the material and time were important in structuring the microbial community during the early stage of decomposition of litter from *Acacia* and *Eucalyptus*. However, the decomposition rate was influenced most by the origin of the material and the incubation site, with no influence or adaptation of the microbial diversity or structure.

## Data availability statement

The datasets presented in this study can be found in online repositories. The names of the repository and accession number can be found in the article.

## Author contributions

CTCCR, FCB, RSP, GMC, JMT, and AR: study conception and design. CTCCR, ESF, HEJ, and FMS: field experimentation. CTCCR, HEJ, ESF, EHN, HEM, and FMS: laboratory procedures. CTCCR: bioinformatic and statistical analysis. CTCCR, FCB, RSP, JMT, EHN, GMC and ASR: drafting of the manuscript. All authors contributed to the article and approved the submitted version.

## Funding

This study was financed in part by the Coordenação de Aperfeiçoamento de Pessoal de Nível Superior-Brasil (CAPES)-Finance Code 001, Fundação Carlos Chagas Filho de Apoio à Pesquisa do Estado do Rio de Janeiro (FAPERJ) and Conselho Nacional de Desenvolvimento Científico e Tecnológico (CNPq).

## Conflict of interest

The authors declare that the research was conducted in the absence of any commercial or financial relationships that could be construed as a potential conflict of interest.

## Publisher’s note

All claims expressed in this article are solely those of the authors and do not necessarily represent those of their affiliated organizations, or those of the publisher, the editors and the reviewers. Any product that may be evaluated in this article, or claim that may be made by its manufacturer, is not guaranteed or endorsed by the publisher.
