# Supplementary material for: Mycobiome structure does not affect field litter decomposition in Eucalyptus and Acacia plantations
Source: Front Microbiol. 2023 Feb 28;14:1106422. doi: 10.3389/fmicb.2023.1106422 (PMC10011713; doi:10.3389/fmicb.2023.1106422)
Supplement: Supplementary file 1 [file Data_Sheet_1.docx]

**Supplemental Material**

**Mycobiome structure does not affect field litter decomposition**

Caio TCC Rachid^1*^, Fabiano C. Balieiro^2^, Raquel S Peixoto^1^, Eduardo da Silva Fonseca^1^, Hugo E. Jesus^1^, Etelvino H Novotny^2^, Guilherme M. Chaer^3^, Felipe M Santos^4,5^, James M. Tiedje^6^, Alexandre S Rosado^1^

1 – Universidade Federal do Rio de Janeiro, Rio de Janeiro, 21941-590, Brazil;

2 - Embrapa Solos, Rio de Janeiro, 22460-000, Brazil;

3 - Embrapa Agrobiologia, Seropédica, 23891-000, Brazil;

4 - Universidade Federal Rural do Rio de Janeiro, Seropédica, 23891-000, Brazil.

5 - Rede ILPF, Brasília, 70.330-530, Brazil;

6 - Center for Microbial Ecology, Michigan State University, East Lansing, 48824, United States of America.

Supplemental Table 1 - Network metrics from *Eucalyptus* and *Acacia* fungal community

|  | ***Eucalyptus* Network** | ***Acacia* Network** |
| --- | --- | --- |
| **Number of nodes** | **120** | **129** |
| **Number of Edges** | **631** | **660** |
| **Average no. of Neighbors** | **11.626** | **10.917** |
| **Network diameter** | **9** | **11** |
| **Network radius** | **5** | **6** |
| **Characteristic path length** | **3.273** | **3.389** |
| **Clustering Coefficient** | **0.426** | **0.450** |
| **Network density** | **0.110** | **0.0992** |
| **Network heterogeneity** | **0.865** | **0.853** |
| **Network centralization** | **0.244** | **0.249** |
| **Connected Components** | **6** | **5** |


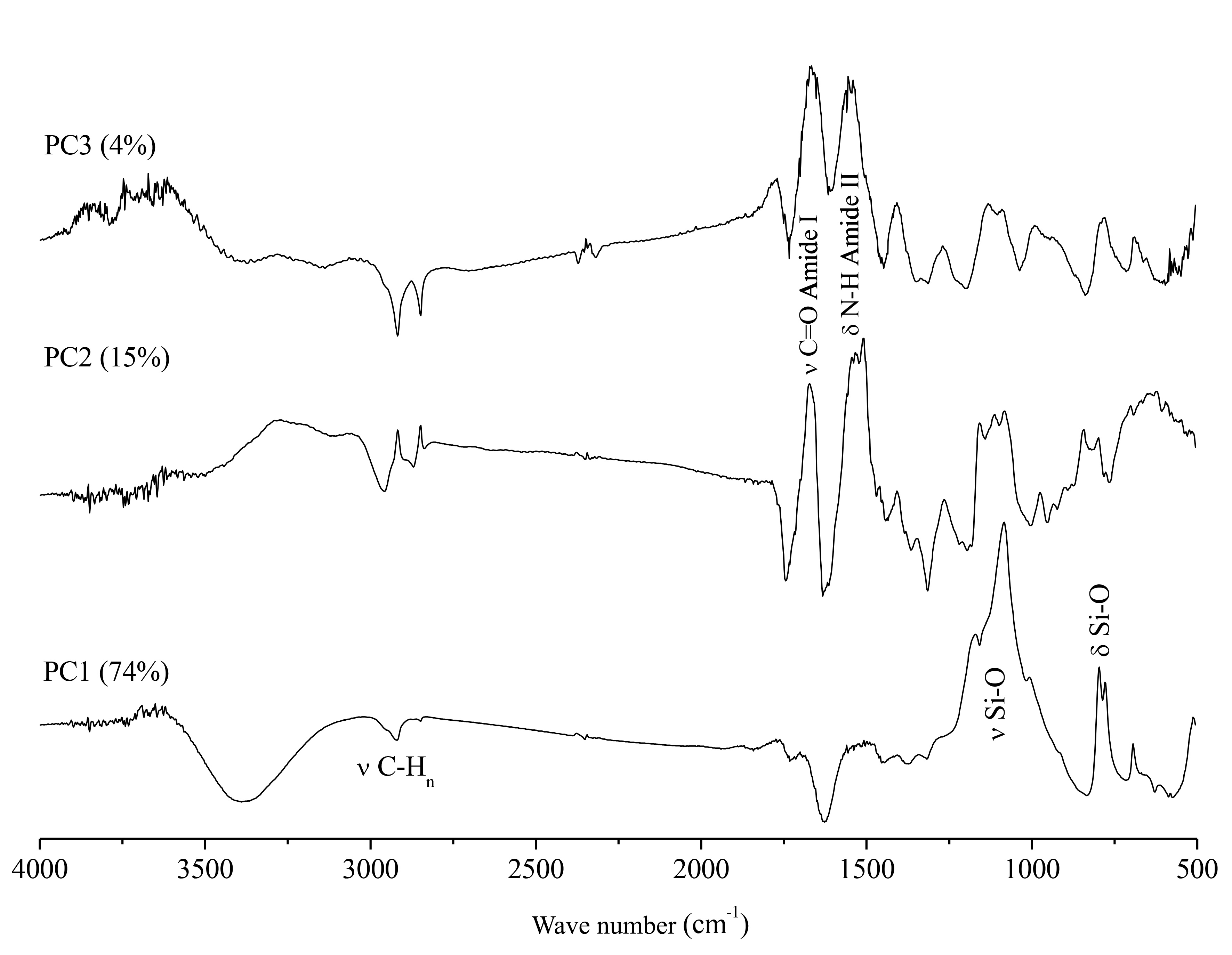


Fig S1. Loading of the first 3 principal component obtained by infrared spectroscopy (FTIR) from litter samples in the treatments Eucalyptus, Mix and Acacia in the different locals of incubation. The 3 components accounted to 93% of all variation. ν: stretch; δ: angular deformation


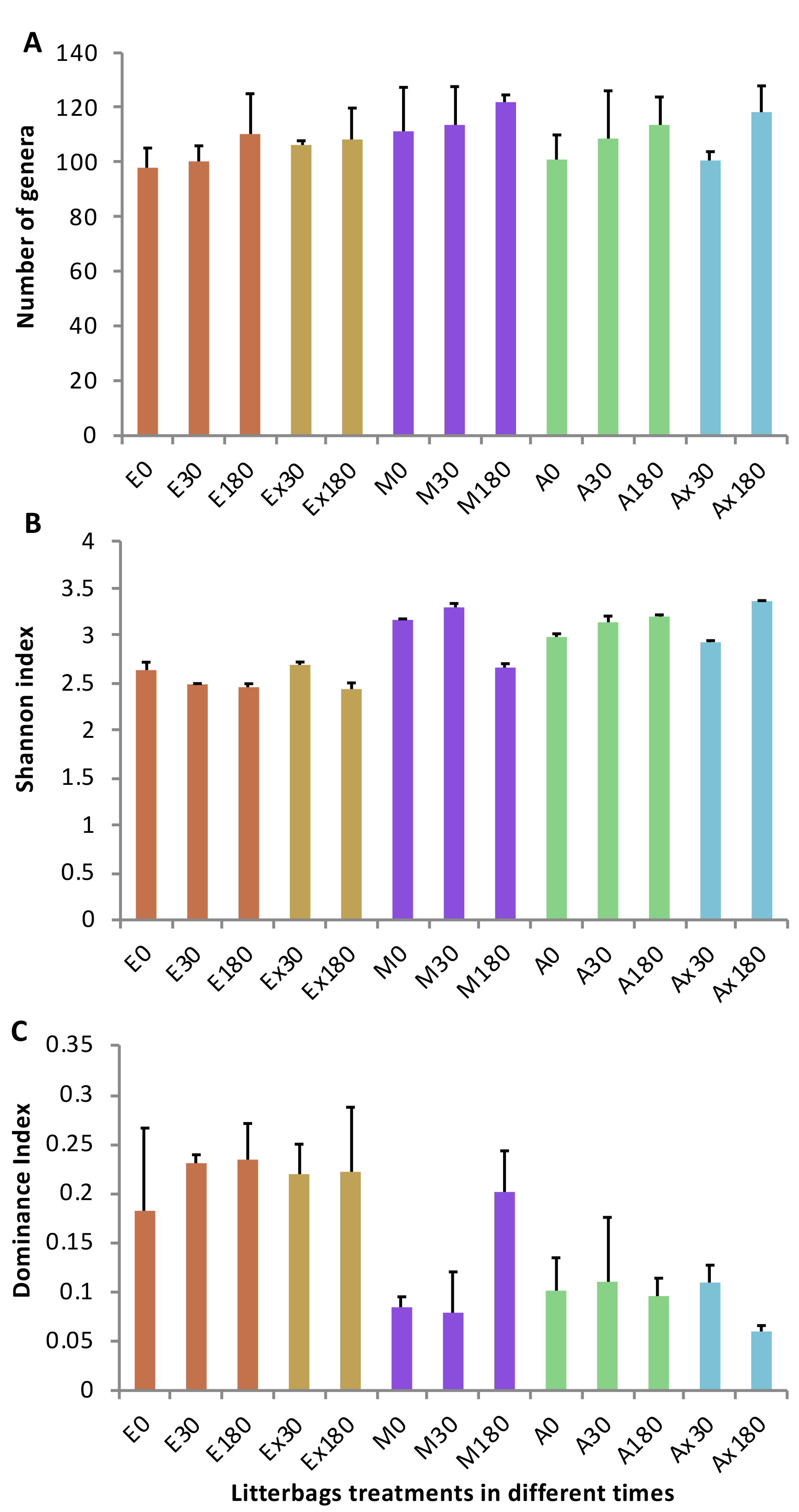


Fig S2. Relative mean (n = 3) values of Richness and diversity indexes of fungi community in samples of litter in different treatments. Initial letters represent the 5 treatments (E – *Eucalyptus*; Ex – *Eucalyptus* X; M – Mix; A – *Acacia*; Ax – *Acacia* X); numbers represent the incubation time in days.


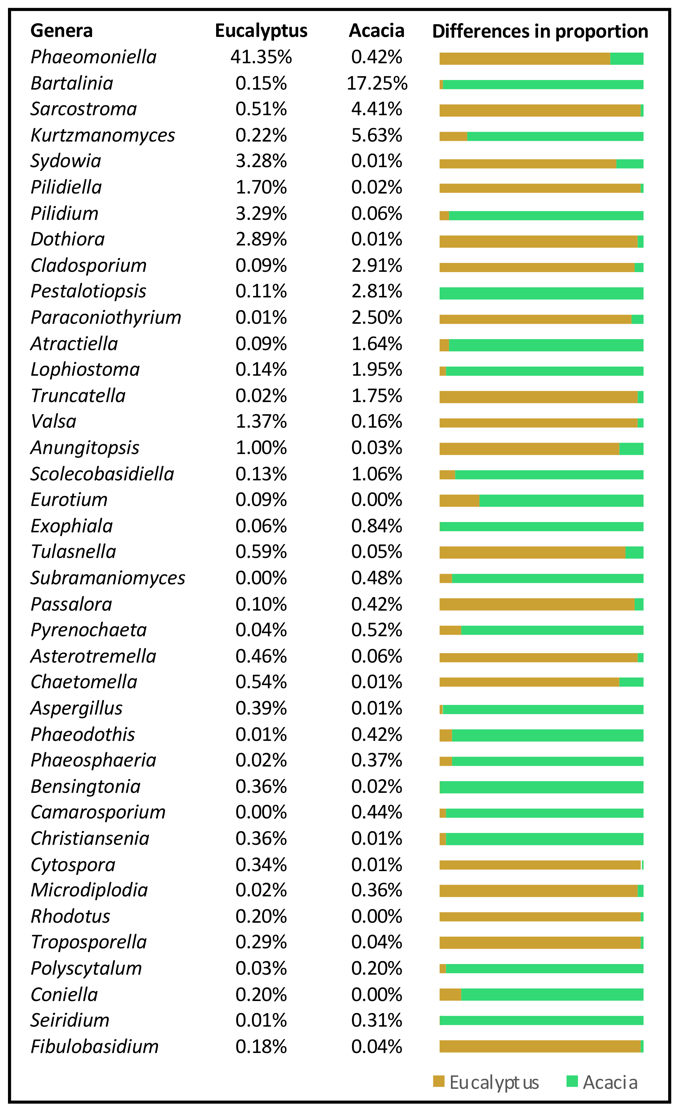


Fig S3. List of the 39 genera of fungi, from the 60 most abundant, which were significantly more abundant in Eucalyptus or in Acacia, according to a blocked indicator species analysis (ISA) performed to compare the composition of the fungal community based on the origin of the litter material.


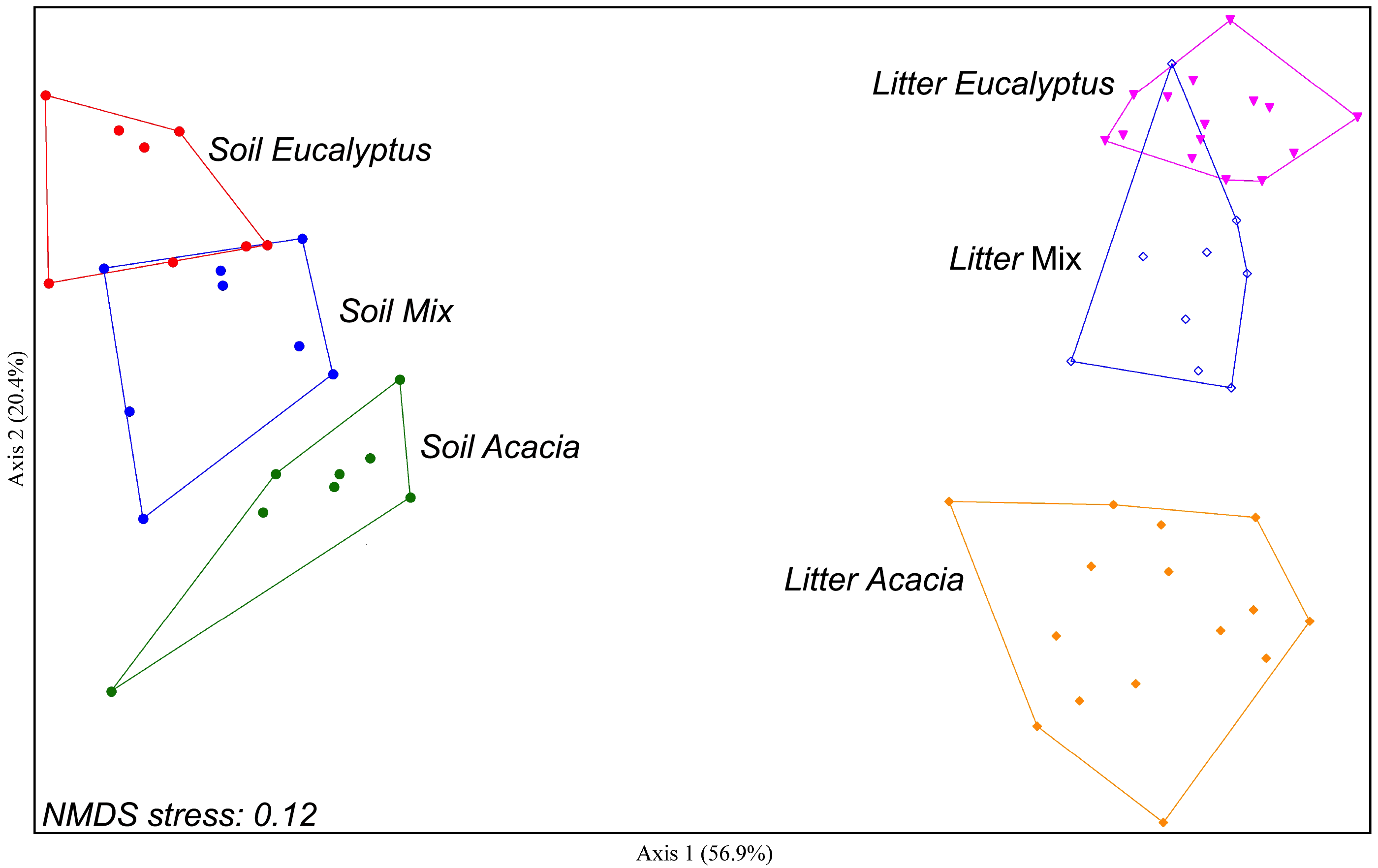


Fig. S4. NMDs ordination of fungal genera profile found in litter samples and in the soil samples where litter were incubated.
